# Boosting efficiency in a clinical literature surveillance system with LightGBM

**DOI:** 10.1371/journal.pdig.0000299

**Published:** 2024-09-23

**Authors:** Cynthia Lokker, Wael Abdelkader, Elham Bagheri, Rick Parrish, Chris Cotoi, Tamara Navarro, Federico Germini, Lori-Ann Linkins, R. Brian Haynes, Lingyang Chu, Muhammad Afzal, Alfonso Iorio

**Affiliations:** 1 Health Information Research Unit, Department of Health Research Methods, Evidence, and Impact, McMaster University, Hamilton, Ontario, Canada; 2 Department of Medicine, McMaster University, Hamilton, Ontario, Canada; 3 Department of Computing and Software, McMaster University, Hamilton, Ontario, Canada; 4 School of Computing and Digital Technology, Birmingham City University, Birmingham, United Kingdom; Wake Forest University School of Medicine, UNITED STATES OF AMERICA

## Abstract

Given the suboptimal performance of Boolean searching to identify methodologically sound and clinically relevant studies in large bibliographic databases, exploring machine learning (ML) to efficiently classify studies is warranted. To boost the efficiency of a literature surveillance program, we used a large internationally recognized dataset of articles tagged for methodological rigor and applied an automated ML approach to train and test binary classification models to predict the probability of clinical research articles being of high methodologic quality. We trained over 12,000 models on a dataset of titles and abstracts of 97,805 articles indexed in PubMed from 2012–2018 which were manually appraised for rigor by highly trained research associates and rated for clinical relevancy by practicing clinicians. As the dataset is unbalanced, with more articles that do not meet the criteria for rigor, we used the unbalanced dataset and over- and under-sampled datasets. Models that maintained sensitivity for high rigor at 99% and maximized specificity were selected and tested in a retrospective set of 30,424 articles from 2020 and validated prospectively in a blinded study of 5253 articles. The final selected algorithm, combining a LightGBM (gradient boosting machine) model trained in each dataset, maintained high sensitivity and achieved 57% specificity in the retrospective validation test and 53% in the prospective study. The number of articles needed to read to find one that met appraisal criteria was 3.68 (95% CI 3.52 to 3.85) in the prospective study, compared with 4.63 (95% CI 4.50 to 4.77) when relying only on Boolean searching. Gradient-boosting ML models reduced the work required to classify high quality clinical research studies by 45%, improving the efficiency of literature surveillance and subsequent dissemination to clinicians and other evidence users.

## Author summary

With so many health-related research studies being published, it can be overwhelming to find the best ones for making healthcare decisions. For nearly 25 years, our research group has been helping by creating tools to search through these studies. Our expert team uses these tools to find and assess the most reliable and important studies for healthcare providers and patients. In this study, we used an automated machine learning technique to speed up this process and reduce the effort needed. We trained models to identify studies with strong methods using data from almost 98,000 articles. We tested over 12,000 models and chose the best one, that used a technique called LightGBM, which missed the fewest good studies. Adding this model to our process reduced our workload by 45%. This means we can now find high-quality research more quickly and efficiently, helping healthcare providers and other users get the best evidence faster.

## Introduction

The identification of high-quality clinical literature is crucial for clinical practice and research, especially considering the increasing pace with which medical literature is produced. There have been multiple approaches taken to support easier information retrieval, extraction, and assessment to assist in the practice of evidence-based medicine. Early approaches to filtering high-quality, clinically relevant articles from those not ready for clinical practice included validated text-based search strategies that filter articles by research methods, such as systematic reviews [1] and randomized controlled trials (RCTs) [2]. These were the foundation of Clinical Queries which have been integrated into biomedical databases, such as PubMed, to improve the efficiency of finding evidence for over 20 years [3]. However, the task of critical appraisal is a growingly complicated and time-consuming process in the evolving field of evidence-based medicine, and the mere identification of study design is no longer sufficient. Currently, the manual critical appraisal process initially involves identifying publication types and study designs and then the appropriate tool to assess transparent reporting and methodological rigor. These tools are also becoming progressively comprehensive. For instance, the revised Cochrane risk of bias (RoB) 2 tool to assess RCTs in systematic reviews involves up to 28 questions per outcome per RCT [4]. The official guideline for the tool is over 70 pages, detailing the rationale behind every decision. In contrast to the original RoB tool, with only seven questions per RCT, RoB 2 represents a significant increase in complexity.

Advancements in machine learning (ML) and natural language processing (NLP) are improving the efficacy and efficiency of evidence curation, extraction, and summarization. There has been considerable attention paid to the production of systematic reviews, with ML models derived to semi-or fully-automate article screening [5–8], and increasingly to information extraction and database searching [8]. The use of ML to identify high-quality studies and to automate the risk of bias and relevance ranking is promising [8]. A 2021 systematic review identified ten articles that applied ML to retrieve high-quality evidence using gold standard datasets of articles that were critically appraised for methodologic rigor [9]. Several studies have assessed the performance of deep learning on rigor assessment [10–12]. We recently published the results of a deep learning BioBERT-based model trained to identify high-quality studies that maintained recall at >99% and improved specificity to >60% [13]. Previous literature also compared deep learning neural networks with shallow learning algorithms on text classification, in which their comparative performance varies greatly depending on the context [14–16]. While neural networks may offer competitive or superior performance without the need for meticulous feature engineering, several important concerns limit their applicability. Their computational demands are prohibitive, as a neural network on a large corpus can take days or weeks to train and fine-tune, even on graphics processing units specifically designed for ML training [17]. The “black-box problem” poses additional challenges for deep learning models [18]. The lack of interpretability and explainability of how neural networks come to their decisions undermines their trustworthiness and ethical use and severely limits their external applications [19]. A clinician would not be able to trace the decision and rationale of an RCT’s rigor rating by a neural network whereas they could with the RoB 2 tool. Numerous methods to explain decisions by deep learning models have been explored [20,21], but the majority are model-specific and focus on tabular or image data as opposed to free text.

In contrast with deep learning methods, shallow learning algorithms are less computationally demanding and can achieve a reasonably high accuracy while minimizing training time. Shallow learning methods are also more interpretable than deep learning models due to their relative simplicity [20,21]. While the interpretation may not be as straightforward as free text rationale provided by human critical appraisal experts, explicit definitions of features and weights enable the examination of factors that contributed to the final decision. Additionally, shallow learning with proper feature engineering may not necessarily perform worse than deep learning models on NLP tasks [14–16,22]. Several articles detailed the use of shallow learning models to appraise methodological rigor [23–29]. Regrettably, these studies suffer from important methodological limitations. Specifically, all of them utilized imbalanced datasets without resampling or incorporating class weights into their models [23–29]. This may limit the learning from minority classes and bias the model decision towards the majority class [30]. Additionally, they focused on a select few models and hyperparameter combinations [23,25,26,28,29], and often did not examine the performance of ensemble models [24–26,29]. Lastly, some studies focused on type [26] or field specific [25] articles, or articles published before 2010 [23,25,27–29], which undermines the generalizability of their models.

**Fig 1:**
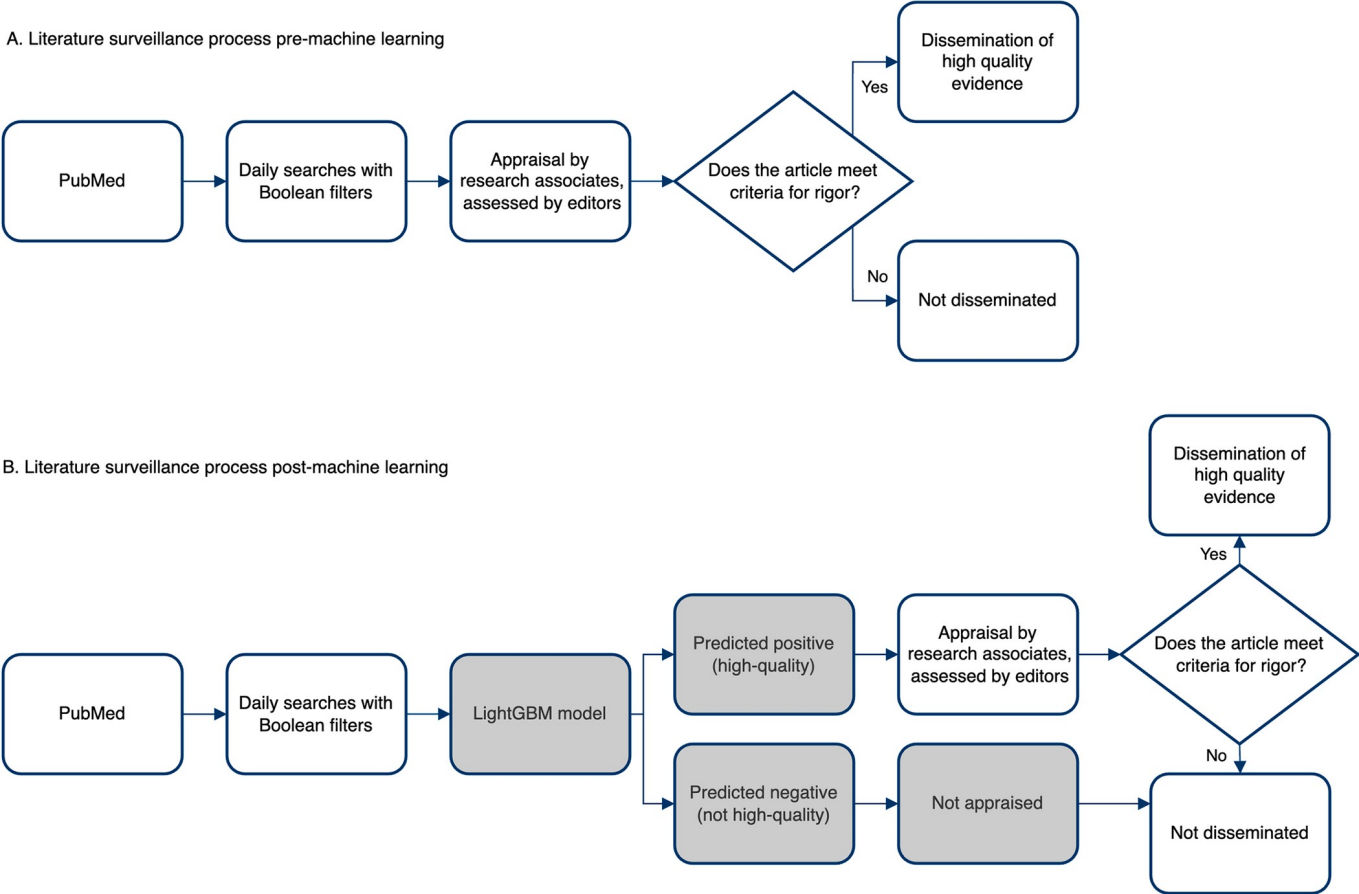
Illustration of the literature surveillance process A. before and B. after addition of a machine learning algorithm to predict quality of the article.

At the McMaster Health Research Information Unit (HiRU), we accelerate access of evidence-based information for practicing clinicians by evaluating literature at the time of publication through the Premium LiteratUre Service (PLUS) (Fig 1A) [31]. Study methods are evaluated using the same criteria as the Hedges dataset that was used to derive Clinical Queries search strategies [32]. In brief, studies published in ~120 clinical journals are retrieved from PubMed using sensitive Boolean search strategies each day and are manually appraised by trained research associates. Articles are assessed for scientific merit based on design-specific criteria, such as random allocation, follow-up, and reporting of clinically important outcomes for RCTs [33]. Articles meeting appraisal criteria are reviewed by clinician editors and rated by practicing clinicians for clinical relevance and newsworthiness [34]. Through PLUS, we have curated a database of articles manually classified according to methodological rigor and clinical relevance since 2012 and has expanded to include all COVID-19 articles from PubMed since March 2020.

As previously mentioned, maintaining a large corpus of appraised clinical articles such as PLUS is a resource-intensive activity. Maintaining high recall (sensitivity >99%) while reducing the number needed to read (NNR)—a measure of human effort required during the critical appraisal step to identify a relevant article—is important in improving the efficiency of this process. This need for efficiency gains is tempered with a mission to maximize inclusion of high-quality clinically relevant studies to maintain a resource that is useful for clinicians and researchers.

To address the limitations of previously published shallow learning methods, we opted for the Microsoft Automated machine learning (AutoML) service to discover optimal models from a multitude of experiments with various parametric combinations. AutoML are tools that iterate, select, and optimize ML models at multiple steps of the process by automating the selection of promising algorithms, hyperparameter tuning, pre-processing, and features selection [35,36]. The systems search through possible model and hyperparameter configurations and select those that perform best on the given task. This reduces the time needed to train and test models and inaccuracies in the model that may arise from human errors and bias.

Objective: To develop and evaluate ML models to improve the efficiency and accuracy of identifying high-quality clinical literature by leveraging the Microsoft AutoML service and resampling methods to maintain high recall (sensitivity >99%) and reduce the number needed to read (NNR) to find one high quality study.

## Materials and methods

We performed a retrospective study using a labelled dataset of articles that were critically appraised for methodologic rigor and clinical relevance to train, validate, and test algorithms that predict the likelihood of a clinical article meeting appraisal criteria for rigor. We used autoML as an efficient approach to training multiple models. Selected models were prospectively evaluated by having trained research associates, blinded to model predictions, appraise incoming articles in the literature surveillance program, as a test of the external validity of model predictions.

### Quality standard database

We define high-quality or rigor as meeting all critical appraisal criteria for a particular article type (review, guideline, original study) or purpose category (treatment, diagnosis, prognosis, etiology for harm primary prevention, quality improvement, economics, or clinical prediction guides) based on established evidence assessment criteria [33]. The manual critical appraisal step has previously documented high inter-rater agreement (kappa > 0.80 for all categories) [37]. Over the course of two decades, we have reviewed more than 500,000 articles and have curated an internal database that includes articles that did not meet methodological rigor criteria or clinical relevance. Notably, the database is unbalanced, with about 4.5 times the number of articles that fail to meet methodologic rigor or clinical relevance than those that pass. The growing database now includes articles on COVID-19 indexed in PubMed not limited to the core journal set. For the task of classifying articles in a binary assessment of meeting or not meeting criteria for methodologic rigor and clinical relevance, we used the titles and abstracts of 97,805 articles of all types and categories mentioned above that were published between 2012–2018. Of these, 17,824 met criteria for rigor for one or more article categories and for clinical relevance; 79,981 did not.

### Model training and performance

**Fig 2:**
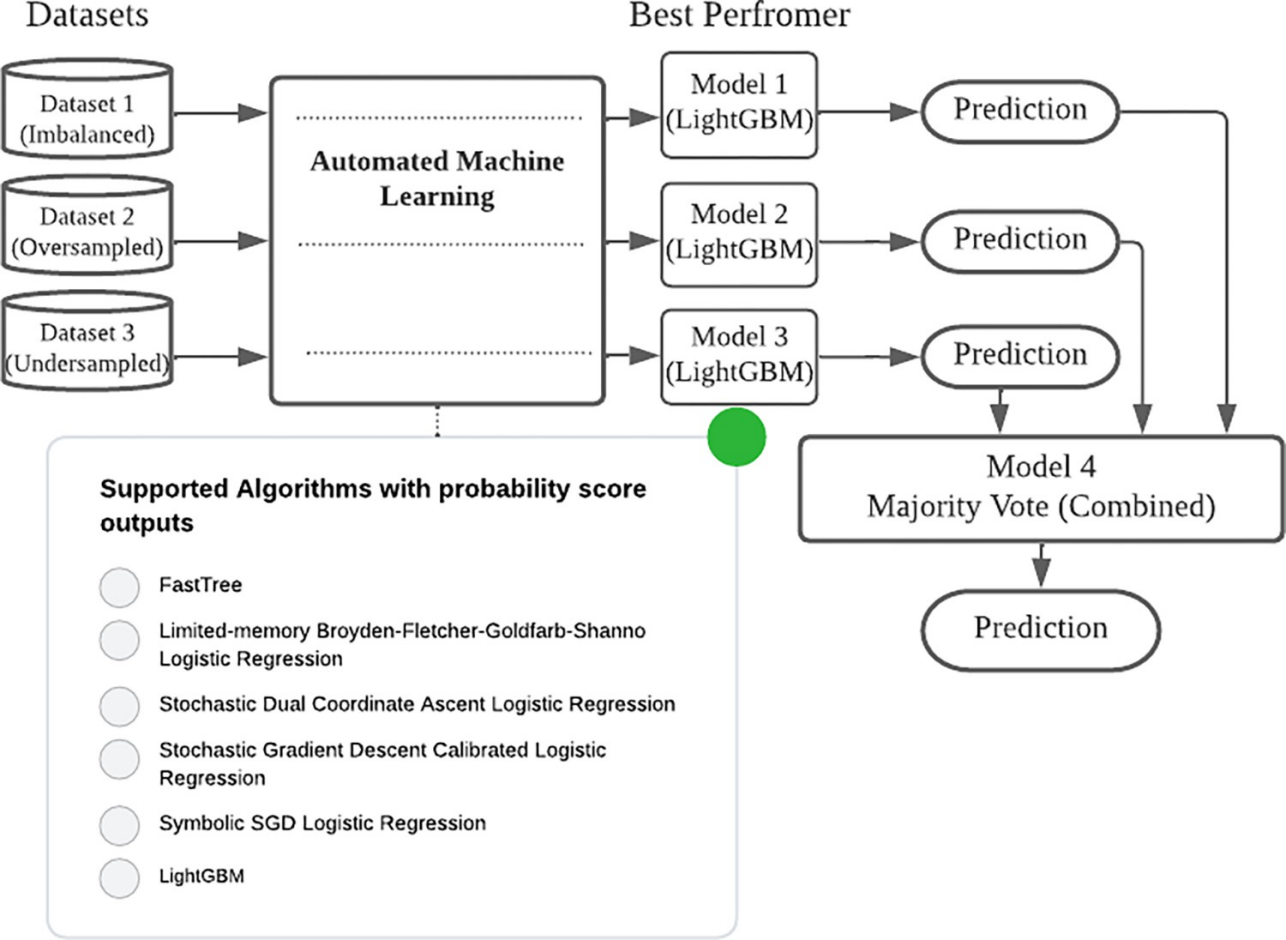
Example depiction of the autoML process.

Our approach to model training was to use AutoML to run multiple sequential experiments with varying settings. The process, depicted in Fig 2, automatically iterated model training using the combinations of pre-processing options, weighting methods, feature selection, and hyper-parameters listed in Table 1, and optimized selections to identify the best performing combinations—essentially the approach optimizes performance and abandons steps that do not lead to better performing models. The performance of an AutoML system depends on the quality of the data and the specific task at hand. We chose AutoML for this study since our dataset was of high quality as it was reviewed and appraised by human experts, and we wanted to remove our biases and grow our understanding of the best approaches for our dataset. AutoML allowed for experimentation while developing expertise. We used Microsoft’s ML.NET AutoML [38] to train and test binary classification models that predicted if an article was of high-quality or not to help identify a highly optimized model, driven by a set goal of improving specificity while maintaining sensitivity above 99%.

We tested weighting by term frequency (TF), inverse document frequency (IDF), and TF-IDF to account for frequency of words within titles and abstracts of articles and their frequency across a dataset. A convenience sample of algorithms available in the public domain and in ML.NET that provided a probability score as an output measure was selected for training. This allowed us to set a threshold of 99% sensitivity rather than the default 50%. The available algorithms at the time of training were FastTree [39], Limited-memory Broyden-Fletcher-Goldfarb-Shanno Logistic Regression [40], Stochastic Dual Coordinate Ascent Logistic Regression [41], Stochastic Gradient Descent Calibrated Logistic Regression [42], Symbolic SGD Logistic Regression [43], and Light Gradient Boosting Machine (LightGBM)[44].

**Table 1:** Parameters and features used in the training of models using automated ML.

Preprocessing/featurization	Options	Datasets applied to
Case	Lowercase or unchanged	All
Numbers	Removed or left as is	All
Punctuation	Removed or left as is	All
Stop words	Removed or left as is	All
Normalization	L1, L2, infinity, or none	All
Ngram length	1 or 2	All
	3	Undersampled dataset only
All lengths*	Yes or no	All
Weighting	TF, IDF, or TF-IDF	All

IDF = inverse document frequency; TF = term frequency.

*All lengths applies when ngram length is >1 and indicates whether it only uses ngrams of the specified length (use all lengths = false) or uses ngrams of all lengths up to and including the specified length (use all lengths = true).

Models with >99% sensitivity were ranked by maximal specificity with the goal of minimizing false positives without missing potentially relevant articles. The classification models were trained using titles and abstracts of a random 80% of the articles (n = 97,805). To address the imbalance in articles, we created 3 training datasets: 80% of the full dataset (unbalanced; n = 97,805), and two additional datasets to achieve balance through oversampling (articles meeting criteria were included multiple times to equal the number of articles that did not; n = 159,962) and undersampling (random subset of articles not meeting criteria were matched to the number that did; n = 35,648).

Trained models were tested on the remaining hold-out set of 20% (n = 24,678) of articles from 2012–2018. Models with ≥99% sensitivity with the best specificity for each of the full, over-, and under-sampled datasets were retained, and one model per dataset was selected from the leaderboard. Models return a probability score ranging from 0 (does not meet criteria) to 1 (meets criteria) for each article. The probability threshold was determined as the point where sensitivity was 99%. To determine if ensembling the three models improved performance compared with the individual models, we tested their performance individually and combined—using a majority vote such that articles predicted to pass in ≥2 of the 3 models were classified as ‘pass’ (or classified as ‘fail’ if 0 or 1 model predicted a pass)—in a retrospective sample of 30,424 articles in our dataset that were published in 2020.

The performance of the models in the hold-out test set is akin to internal validation. Since our goal is to implement an algorithm into a literature surveillance program, we assessed its performance in real-time in an external test on unseen data. We prospectively evaluated the performance of the majority vote algorithm by applying it after Boolean searches of PubMed and before critical appraisal by our research associates, who were blinded to the predictions of 5253 articles published between March 9 to May 11, 2021. Staff appraised all articles predicted to pass and a random subset of those predicted to fail. False negative articles were assessed by a senior clinical researcher (RBH) to determine clinical relevance and newsworthiness.

### Evaluation metrics

For all trained models, during the testing phase we calculated sensitivity (recall), specificity, accuracy, precision, NNR (1/precision), and F-score in the 20% hold-out set of articles from 2012–2018. We also calculated the area-under-the-curve (AUC) of the receiver operating characteristic (ROC) curves, calibration curves [45], and work saved over sampling at 99% recall (WSS@99%; the percentage of all articles that are predicted negative by the algorithm and therefore not reviewed) [46]. The statistical probability was calculated for the three selected models and majority vote algorithm in the 2020 data and the prospective evaluation. For the prospective evaluation, we estimated the bias-corrected sensitivity and specificity with corresponding 95% confidence intervals (CIs) using the Begg and Greenes [47] formula that corrects for any bias when only a subsample is verified to account for the articles that were predicted to fail and that were not verified by design. The bias correction models the diagnostic distribution of the articles that were verified [47].

## Results

### Selected models and their performance

We trained 3456 models using each of the unbalanced and oversampled datasets and 5760 models using the undersampled dataset. The preprocessing steps and parameters used in the selected top performing models are shown in Table 2; each of the three selected models used the LightGBM binary classification algorithm [48]. LightGBM is a gradient boosting framework that uses decision tree algorithms. It is a more efficient implementation of gradient boosting decision tree [44,49] which is an ensemble model of decision trees trained in sequence and a widely-used machine learning algorithm due to its efficiency, accuracy, and interpretability. The performance characteristics of each of the three models in the test datasets from 2012–18 and 2020 are listed in Table 3. The oversampled dataset shows more variation in the ROC curves of all trained classifiers while the classifiers trained on undersampled data also have slightly more variation in performance compared to unbalanced data (S1 Appendix). The AUC values for the three top performing models were very close to each other indicating a high performance for the selected LightGBM model in all three cases. The calibration curves (S2 Appendix) indicate that the unbalanced model is well calibrated and the other two models overestimate the positive class, resulting in more false positives [45].

**Table 2:** Characteristics of the dataset, preprocessing, and feature extraction steps employed by AutoML in the training of the model selected from each dataset experiment*.

	Model 1 (Unbalanced dataset)	Model 2 (Balanced by over-sampling)	Model 3 (Balanced by under-sampling)
Number of articles in training datasets	97,805	159,962	35,648
Ratio of negative:positive articles (or %positive)	4.5:1	1:1	1:1
Number of models trained	3456	3456	5760
Features employed in the selected best model:			
Text converted to lowercase	Yes	Yes	No
Removal of punctuation	Yes	Yes	Yes
Removal of stop words	Yes	No	No
Removal of Diacritics	Yes	Yes	Yes
Removal of numbers	Yes	Yes	Yes
Weighting method	TF-IDF	TF-IDF	TF-IDF
Normalization technique	None	None	L1
N-grams	Uni-grams	Bi-grams	Tri-grams

L1 = Manhattan Distance or Taxicab norm. TF-IDF = term frequency—inverse document frequency.

*All selected models used LightGBM binary classification model.

**Table 3:** Performance characteristics for the three models in the testing datasets (20% from 2012–2018, and 2020).

Testing dataset	Model	Sensitivity (95% CI)	Specificity (CI)	Precision	F-score	Accuracy	NNR (CI)	AUC (CI)	WSS@99%
2012–2018*	Over-sampled	99.0% (98.7 to 99.3)	53.7% (53.0 to 54.4)	32.6%	0.490	62.1%	3.07 (3.02 to 3.13)	0.952 (0.949 to 0.956)	43%
	Unbalanced	99.0% (98.7 to 99.3)	51.0% (50.3 to 51.6)	31.3%	0.475	59.8%	3.20 (3.14 to 3.26)	0.952 (0.949 to 0.955)	41%
	Under-sampled	99.0% (98.7 to 99.3)	51.8% (51.1 to 52.5)	31.6%	0.480	60.5%	3.16 (3.10 to 3.22)	0.948 (0.944 to 0.951)	41%
2020†	Combined‡	99.2% (98.8 to 99.4)	57.5% (56.9 to 58.1)	26.2%	0.415	63.0%	3.86 (3.79 to 3.93)	NA	49%
	Over-sampled	99.1% (98.7 to 99.4)	57.3% (56.7 to 57.9)	26.1%	0.413	62.8%	3.87 (3.80 to 3.95)	0.962 (0.959 to 0.964)	49%
	Unbalanced	99.0% (98.7 to 99.3)	56.4% (55.8 to 57.0)	25.7%	0.408	62.0%	3.94 (3.86 to 4.01)	0.959 (0.956 to 0.962)	48%
	Under-sampled	99.0% (98.7 to 99.3)	56.0% (55.4 to 56.6)	25.5%	0.406	61.7%	3.96 (3.88 to 4.04)	0.956 (0.953 to 0.959)	48%

NA = not applicable; NNR = number needed to read; WSS@99% = work saved over sampling at 99% recall.

*20% of the articles from 2012–2018 for internal testing, n = 24,677

†n = 30,424

‡Predictions determined by majority vote of articles meeting 2 of 3 probability thresholds from the unbalanced, over- and under-sampled models to pass.

### Prospective evaluation

**Fig 3:**
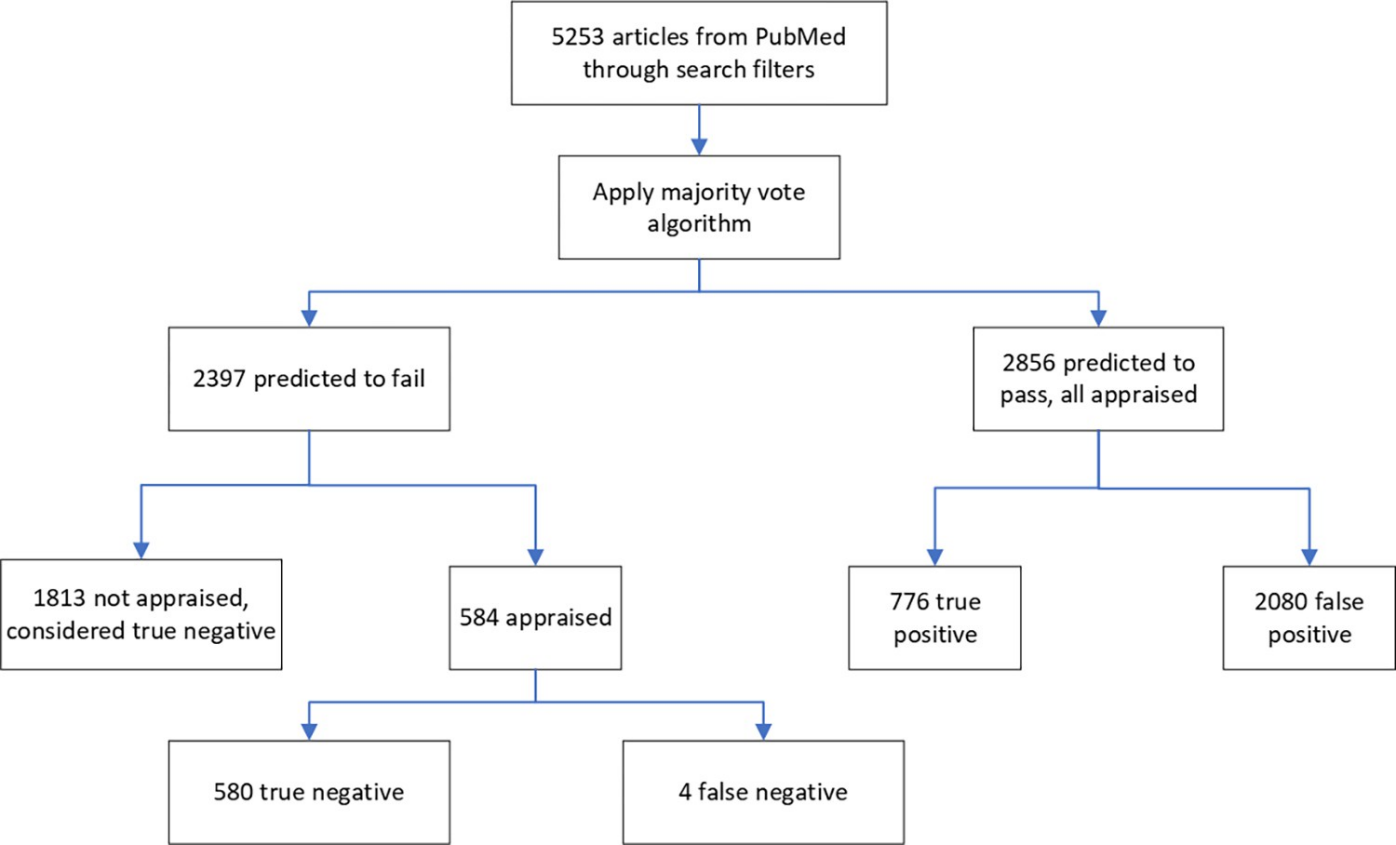
Prospective evaluation of model performance in >5000 articles retrieved from PubMed.

For the prospective evaluation, we opted to use the majority vote algorithm to classify 5253 consecutive articles entering the surveillance system; 2856 (54%) were predicted to be high quality and 2397 (46%) were not (Fig 3). All the 2856 predicted to be high quality and a random sample of 584 of the 2397 predicted to not be high quality were assessed by human appraisers. The remaining 1813 (90%) were not assessed and considered true negatives. Of the random sample predicted to not be high quality and appraised by staff, four were adjudicated to be high quality (false negatives), all of which required using information from the full-text of the manuscript to confirm they met the appraisal criteria for their article categories. Sensitivity was 99.5% (CI, 98.7 to 99.9), specificity was 53.5% (CI, 52.0 to 55.0), and the F-score was 0.427 (Table 4). The results of the corrected analysis that adjusts for 1813 articles that were not assessed (bias corrected calculation) overlapped with the uncorrected values (Table 4).

**Table 4:** Prospective performance of the majority vote ML algorithm.

Article subset	N	Sensitivity (95% CI)	Specificity (CI)	Precision	F-score	Accuracy (balanced accuracy)*	NNR (CI)	WSS@99%
**All**	5253	99.5% (98.7 to 99.9)	53.5% (52.0 to 55.0)	27.2%	0.427	60.3% (76.5%)	3.68 (3.52 to 3.85)	45%
**All-corrected†**	5253	97.9% (95.9 to 99.9)	53.4% (51.3 to 55.4)	NA	NA	NA	NA	NA
**COVID**	3317	100% (97.4 to 100)‡	59.3% (57.6 to 61.1)	9.7%	0.177	61.0% (79.7%)	10.29 (9.33 to 11.49)	57%
**Non-covid**	1936	99.4% (98.8 to 100)	39.2% (36.5 to 41.8)	44.7%	0.617	59.1% (69.3%)	2.24 (2.11 to 2.37)	26%

NA = not applicable; NNR = number needed to read; WSS@99% = work saved over sampling at 99% recall.

*Balanced accuracy = (sensitivity + specificity)/2.

†Bias correction to account for the 1813 articles that were predicted to fail but not verified [47].

‡97.5% one-sided CI.

## Discussion

### Model training and performance

The approach of using AutoML and supervised machine learning led to efficient development of models for identifying articles pre-filtered by highly sensitive Boolean searches likely to be found rigorous and clinically relevant at critical appraisal. Adopting AutoML was time efficient, with models trained using a CPU and most of them trained within 1–5 minutes and allowed for the system to test various permutations of preprocessing steps and algorithms with minimal programmer time. Each of the selected highest performing models used the LightGBM binary classification algorithm, which is computationally a fast algorithm, shown to speed up training by 20 times, while maintaining accuracy [44].

Training the models with datasets of varying size and balanced/unbalanced data allowed us to assess the value in data augmentation. We also explored the effect of combining models to determine if such an approach would improve performance. Though the improvement was very small, our decision to test the ensemble and implement it was based solely on our efforts to maximize specificity to reduce the NNR. Keeping sensitivity high at 99%, the specificity of the trained models was >50% in the random test set from 2012–2018, with slightly better performance with the model trained using the larger oversampled dataset compared with the unbalanced and undersampled datasets. The calibration curves show the unbalanced model to have the best calibration.

Though oversampling offered a larger sample, it came at the cost of time required for model training and led to overfitting of the model since we replicated positive cases to achieve balance. Despite having more models trained using the undersampled dataset, the performance of the top models was consistent with the unbalanced dataset model. All models had similar specificity in the 2020 dataset and performed marginally better than in the 2012–18 set. This could be the result of a larger sample and a broader range of journal titles and article types with the inclusion of COVID-19 publications.

The results for the majority vote combined models, where articles predicted to pass for at least two of the three models, did not factually improve the performance in the three testing datasets across years. Such ensemble approaches of combining models have been used by Aphinyanaphongs et al., [28] and Kilicoglu et al. [27] and showed improved F-scores. Ensemble techniques are used to reduce variability across models by averaging out the errors made by each, assuming they are making different errors [50]. Ensemble models generally perform better when the base models they combine are as diverse as possible [51]. Our three models were built to represent the full unbalanced dataset, a balanced undersampled dataset, and a larger oversampled dataset, but they include the same positive class of articles employed the same type of ML model and are likely not diverse enough to boost performance when combined.

Testing and application of the ML models improved specificity compared with our traditional approach of Boolean filters alone, but not as well as DL-PLUS, the BioBERT-based model we previously reported [13]. Our goal was to maximize recall/sensitivity and specificity and reduce the NNR using a model that is light weight and efficient. Prior to applying the ML models to the PLUS process (and before COVID-19), our NNR in 2019 was 4.63 (95% CI, 4.50 to 4.77). With the addition of COVID-19 articles, in 2020 our overall NNR was 7.11 (CI, 6.92 to 7.31). In the 2021 prospective evaluation with the addition of the LightGBM majority vote model, the NNR was reduced to 3.68 (CI, 3.52 to 3.85) for all article categories with WSS@99% of 45%. In the DL-PLUS prospective validation, the NNR across all articles was reduced to 3.0 (CI, 2.8 to 3.1), with 63% WSS@99%. NNR, measured as 1/precision, is driven also by the proportion of articles, as reflected in the higher number of COVID articles which made up >63% of articles in the prospective evaluation data.

### Machine learning for biomedical evidence

Our approach is consistent with reported methods in our recent systematic review of ML applied to improve the identification of high-quality articles [9]. We used an established gold standard for high quality articles produced through our PLUS process. Seven studies included in the review trained their models using the Hedges dataset or articles included in ACP Journal Club, both of which are produced by the same process in HiRU [9]. Like other studies, we used title and abstracts as training features. Of the 10 studies included in our earlier systematic review, seven used datasets of articles that had been critically appraised by our process [9].

Our models optimized recall to reduce the loss of relevant articles but that came at the cost of reducing specificity and precision. The precision of our models, which ranged from 26% to 33%, was surpassed by Kilicoglu et al. [27] who used ensemble models (74%), and Del Fiol et al. [10] (34%) and Afzal et al. [11] (86%) who used neural network models. For our DL-PLUS model, precision was 42%. The higher precision achieved is likely attributed to the targeting particular categories of articles. Kilicoglu et al. [27] used an ensemble model which achieved a precision of 37% and recall of 63% when applied to articles in general, and precision of 74% and recall of 86% when used to identify rigorous treatment articles—all of which are RCTs—a category with established terminology and structure for reporting. Afzal et al. [11] used the Cochrane library as training dataset for their neural network, which includes systematic reviews and RCTs, which again use explicit study design terminologies in the title, abstract, or commonly both. This facilitates the retrieval function for the model and improves the overall model performance [11]. The use of additional features, like MeSH terms and MEDLINE metadata could also explain the improved performance of their model, though these elements are not readily available for an article when it is first posted in PubMed as there is a delay between PubMed creation date and indexing being applied, which varies by journal title [52]. As we apply our appraisal within 2 weeks of being posted to PubMed, we did not include other potential variables such as citations, which accrue over time after publication, or author etc. Aphinyanaphongs et al. [28,29] trained models using treatment, diagnosis, prognosis, and etiology articles from ACP Journal Club which reflects the range of article types included in our dataset.

Ambalavanan and Devarakonda trained sciBERT, a pretrained deep learning algorithm, and looked at both class ratios and size of the training sets for classifiers of treatment articles using the Clinical Hedges dataset [53]. They found that recall was maximized when there were more positive to negative articles, precision was improved in larger training sets though there appeared to be a point at which having a larger dataset did not result in improved performance, and the F-score was optimal using a reasonably large set of balanced articles (15,000:15,000). They modeled a number of steps in the article classification process (e.g., of interest to humans, original study, treatment article, rigorous), and found that the F-score was lowest for predicting rigor, which is a more difficult task. Notably, their study focused on articles in the treatment category while our model covers articles from the full range of categories covered in the surveillance process.

F-score is the balance between recall (not missing a significant number of instances) and precision (the proportion of relevant instances within the retrieved documents) and it provides an intuitive value of the robustness of the developed models. The article classification tasks assigned to the model were binary, with recall optimized to increase the model robustness over its precision. This intentional optimization towards higher recall was guided by our motive to minimize the chance of losing relevant high-quality articles. This limited our flexibility in maximizing precision and resulted in a lower overall F-score. The wide range of article categories in both training dataset and the stream of articles screened by the model would also have reduced the F-scores. Had we sought to classify articles from a particular purpose category, such as treatment studies using RCT designs, we expect the F-score would be higher.

### Implications for evidence surveillance

Retrieving the best quality evidence for clinicians has driven research into the creation of initial Boolean search strategies and now the advancements made applying ML models. We implemented the majority vote ML algorithm into our process in May 2021 (see Fig 1B). Between May 11, 2021, to Mar 11, 2022, 25 867 articles were retrieved from PubMed with the Boolean searches; 11 776 (45.5%) were predicted not to meet criteria and were removed from the critical appraisal queue. With a conservative estimated time of 5 minutes of human resources to appraise each article, this saved >981 hours of research associate time during that period while maintaining the integrity of the evidence processed. This has been particularly important as we added COVID-19 related articles from all indexed journals to our surveillance program in 2020 to support quick access for practitioners, policy makers, and lay persons to appraised emerging research through the COVID-19 Evidence Alerts website [54]. The LightGBM, and subsequently the DL-PLUS BioBERT models offset some of the additional burden of this growing body of COVID-19 literature as seen in the higher WSS@99% of 63%.

The LightGBM model has been employed to support updates in DynaMed for disease areas with low volume of evidence [55]. To reduce the burden of manual review by topic authors, 91,009 articles retrieved by disease-specific content searches in PubMed were ranked by LightGBM and 8,406 (9.2%) of the highest ranked were manually reviewed for relevance and 576 (6.9%) were used to update 241 topics [55]. This demonstrates another real-world example of ML-supported evidence retrieval and curation.

AI-driven models that can facilitate information retrieval, processing, and summarization can support users who have clinical questions but limited time to search for answers and producers of evidence materials like clinical textbooks, systematic reviews and guidelines [8]. As LLMs continue to evolve and improve, we expect greater efficiency and easy access to evidence based answers.

### Strengths and limitations

Our models were trained using the largest known critically appraised and tagged dataset of health care research articles across a range of article categories to date and based on an established gold standard for rigor in the field [9]. Although the critical appraisal criteria are applied by a single reader, all included studies and those passing with questions are assessed by a final editor and clinical relevance is assigned by practicing clinicians in the related clinical discipline. The dataset overcomes some of the challenges we identified in our review: 1) the criteria applied to assess rigor is an established gold standard based on best evidence-based medicine practices; 2) the dataset is the largest, yet, and the training dataset included 17 824 articles in the high-quality class that allowed for creating oversampled and undersampled datasets for training; 3) journals for a range of clinical domains are included in the dataset [56]; and 4) the training dataset is contemporary and includes articles from 2012–2018 and was tested in 2020 data. The prospective, blinded evaluation of the performance of the selected combined models highlights the value of real-world application and impact.

The models, however, were derived using prefiltered articles from PubMed for a subset of ~120 journals and generalizability to all the content in a literature database is uncertain. These concerns are allayed by the performance of the models in the 2020 articles which are more numerous and cover a greater array of journal titles as all pre-filtered COVID-19 articles were included. Though the number to read was higher (not surprising given the amount of lower quality evidence in COVID-related studies), specificity and accuracy were improved. The calibration curve for the unbalanced dataset suggests a well-calibrated model that maintains accurate probabilities. In contrast, the two balanced models appear to be poorly calibrated, indicating that the balancing process may have introduced bias, leading to the development of suboptimal models. Models were trained using only titles and abstracts of the articles whereas research associates use the full-text of the articles to assess rigor, and the four false negative articles in the prospective evaluation required information beyond the abstract to make an assessment. Since we apply the model to articles as soon as they enter PubMed, we did not use other potential variables in model training, such as keywords, author information, citation networks, or indexing terms as they are not consistently available or reliable or take time to accrue.

### Future model development

In this study, we used logistic regression approaches, and more advanced deep learning techniques perform better [10,11,13]. We plan to evaluate models for applications other than literature surveillance alone and investigate questions about optimal class ratios and training dataset size for model development. Our future research includes assessing model performance by category of articles and applying our models more broadly beyond the journal titles monitored for PLUS. Given the richness of our dataset, including tagged reasons for not meeting critical appraisal criteria and other article metadata captured at the time of appraisal, we hope to enhance model performance by leveraging these data.

The active surveillance program allows for human-in-the loop active training of the models, and exploration of how the models make decisions. This research will help us contribute to a deeper understanding of ML models in this field.

## Conclusion

By employing the boosting method LightGBM, we achieved significant increase in the specificity of identifying biomedical articles that meet methodological rigor criteria and are relevant to clinical practice while preserving a very high sensitivity. The selected models perform well in an active surveillance program that supports knowledge translation to practicing clinicians.

## Supporting information

S1 Appendix Receiver operating characteristic (ROC) curves for the models trained on the 3 datasets. (PDF)

S2 Appendix Calibration curves for the 3 LightGBM models trained using A. undersampled, B. unbalanced, and C. oversampled datasets in the dataset of articles from 2020.
(DOCX)
