# Supplementary material for: Boosting efficiency in a clinical literature surveillance system with LightGBM
Source: PLOS Digit Health. 2024 Sep 23;3(9):e0000299. doi: 10.1371/journal.pdig.0000299 (PMC11419392; doi:10.1371/journal.pdig.0000299)

Appendix A. Receiver operating characteristic (ROC) curves for the models trained on the 3 datasets.

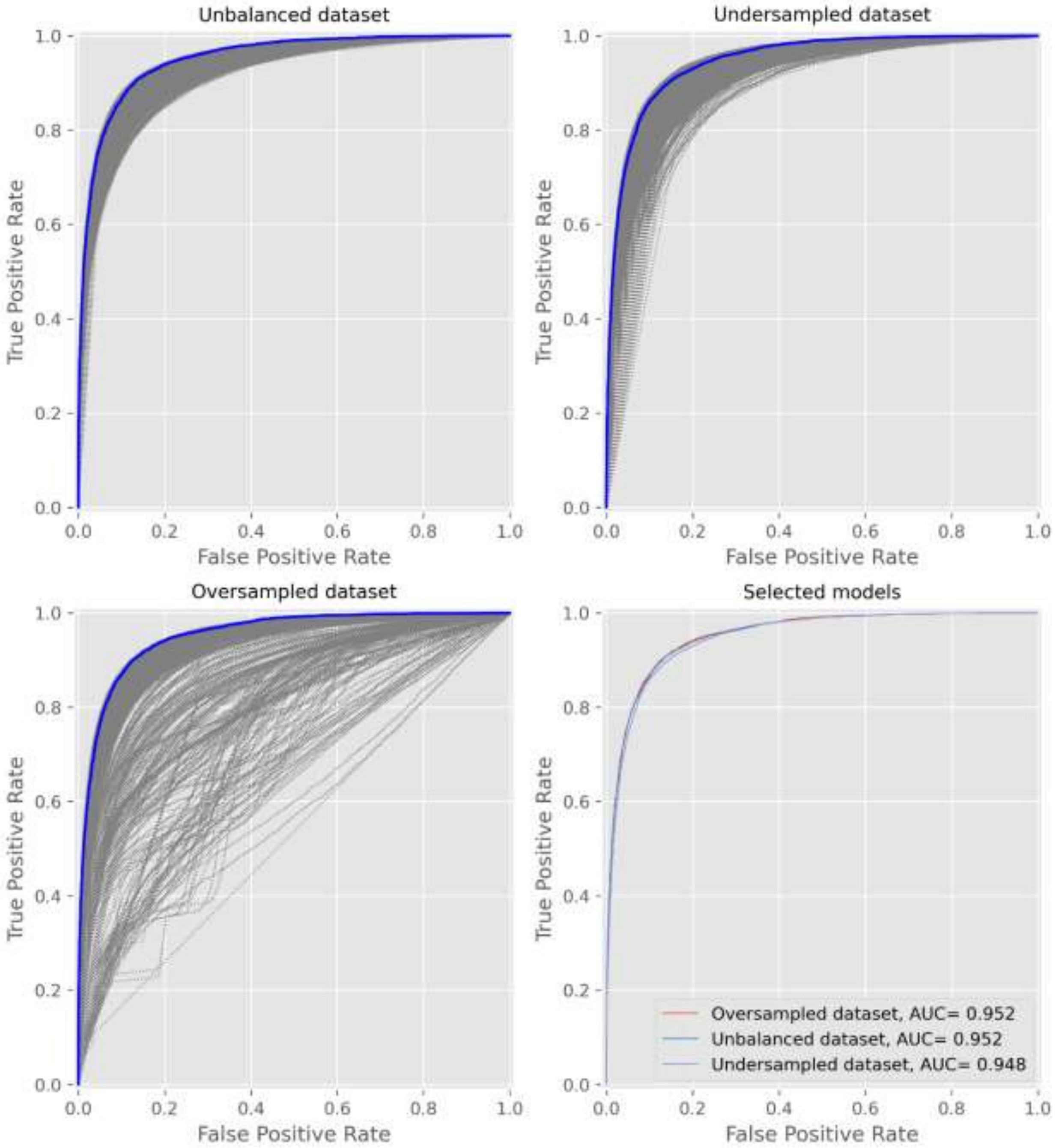

Supplement: S1 Appendix — (PDF) [file pdig.0000299.s001.pdf]
