# Supplementary material for: Boosting efficiency in a clinical literature surveillance system with LightGBM
Source: PLOS Digit Health. 2024 Sep 23;3(9):e0000299. doi: 10.1371/journal.pdig.0000299 (PMC11419392; doi:10.1371/journal.pdig.0000299)
Supplement: S2 Appendix — Calibration curves for the 3 LightGBM models trained using A. undersampled, B. unbalanced, and C. oversampled datasets in the dataset of articles from 2020. (DOCX) [file pdig.0000299.s002.docx]

Appendix B. Calibration curves for the 3 LightGBM models trained using A. undersampled, B. unbalanced, and C. oversampled datasets in the dataset of articles from 2020.
